# Case report: The feasibility of rTMS with intrathecal baclofen pump for the treatment of unresolved neuropathic pain following spinal cord injury

**DOI:** 10.3389/fresc.2022.893014

**Published:** 2022-07-26

**Authors:** Stevie D. Foglia, Ravjot S. Rehsi, Claudia V. Turco, Harsha Shanthanna, Aimee J. Nelson

**Affiliations:** ^1^School of Biomedical Engineering, McMaster University, Hamilton, ON, Canada; ^2^Department of Kinesiology, McMaster University, Hamilton, ON, Canada; ^3^Faculty of Medicine and Dentistry, University of Alberta, Edmonton, AB, Canada; ^4^Faculty of Health Sciences, McMaster University, Hamilton, ON, Canada

**Keywords:** neuropathic pain, repetitive transcranial magnetic simulation, rTMS, baclofen pump, intrathecal pump, spinal cord injury

## Abstract

The main objective of this study was to assess the efficacy and safety of 10 Hz repetitive transcranial magnetic stimulation (rTMS) for the treatment of unresolved neuropathic pain in an individual with spinal cord injury and an intrathecal baclofen pump. A 62-year-old male presented with drug resistant neuropathic pain as a result of a complete spinal cord lesion at T8 level. Pain was classified into four types: pressure pain in the left foot, burning pain in buttocks, burning pain in sternum, and electrical attacks in the trunk. The treatment period involved 6 weeks of rTMS stimulation performed 5 days per week, a 6-week follow up period with no stimulation, and an 8-week top up session period which began 5-weeks after the end of the follow up period. 2004 pulses were delivered at 10Hz over the right-hand representation of the left primary motor cortex at 80% resting motor threshold during each session. Assessments were based on the numerical rating scale (NRS), neuropathic pain scale (NPS), Hamilton Depression and Anxiety rating scales. Following the treatment period there was a 30, 13, and 29% reduction in sternum, buttocks, and left foot pain respectively, as reported by the NRS. During this time, electrical attacks were abolished following the third week of treatment. These changes corresponded to a 38% decrease in NPS scores and a 65 and 25% reduction in anxiety and depressions scores respectively. The changes in sternum, buttocks, and left foot pain reported on the NRS persisted for 1 week following treatment. Top up sessions delivered 11 weeks after the end of the treatment period were unsuccessful in reducing pain to the level achieved during the treatment period. A 13% reduction in NPS was seen during these 8-weeks. Anxiety and depression scores decreased 78 and 67% respectively. The frequency of electrical attacks was zero during this time. rTMS stimulation delivered throughout this study did not cause any interference with the functioning of the intrathecal baclofen pump. This case study illustrates that rTMS may be effective at reducing drug resistant neuropathic pain with certain pain types exhibiting greater propensity for change.

## Case presentation

The participant is a 62-year-old male suffering from NP due to a complete spinal cord injury at T8 that occurred in year 2010. The patient had noted that no medication or other therapy had improved pain symptoms in the past. He had an intrathecal baclofen (ITB) pump therapy introduced in 2017. Subsequently, patient has been tried with several other options including, acupuncture, IV lidocaine therapy, opioids (both oral and intrathecal), gabapentin, and others. During the time of this study, his medications included gabapentin, cymbalta, zopiclone, and oxybutynin. Due to the refractoriness and severity of pain, the patient was referred by the neuromodulation team at Hamilton Health Sciences to take part in this study. The patient suffered from daily prolonged pain in the lower limbs, buttocks, and trunk including burning and electrical shock-like sensations. Four pain phenotypes were identified by the patient and tracked throughout this study. These pain phenotypes began within the first month following the injury and have been consistent for the 11 years prior to beginning the study. These pain phenotypes included:

1. Left foot: Described as a continuous intense pressure that feels like the skin on the foot is going to burst. Rated at baseline as a 7/10 on the numeric rating scale.
2. Sternum: When sternum is touched it causes burning pain in sternum area. Only occurs if sternum is touched. Rated at baseline as a 10/10 on the numeric rating scale.
3. Buttocks: Described as a continuous sharp electrical type of pain/ burning in the lower pelvic tailbone area. Rated at baseline as an 8/10 on the numeric rating scale.
4. Electrical attacks: Described as attacks of sharp circulating electrical pain that would begin in the left flank and radiate throughout the torso (back and core). Would occur 2–3 times per day and would last up to a minute. Rated at baseline as a 10/10 on the numeric rating scale.

## Introduction

Neuropathic pain (NP) is caused by a lesion or disease of the somatosensory nervous system (1, 2). NP dramatically decreases the quality of life for a patient and is associated with severe emotional and physical consequences such as impaired function, depression, sleeplessness, and chronic fatigue (3, 4). Approximately 60% of patients experience NP following spinal cord injury (SCI) (5). Even with evidence-based pharmacological intervention strategies, a 50% reduction in pain is only achieved in one-third of SCI patients (6).

Repetitive transcranial magnetic stimulation (rTMS) offers a potential opportunity to non-invasively treat patients with NP who are refractory to pharmacological intervention. Several sham-controlled studies have demonstrated that rTMS applied to the primary motor cortex has an analgesic effect on NP symptoms in patients with SCI (7–11). This effect has been shown to last up to 6-weeks post treatment (9). Although the duration of treatment intervention varies across studies, the current literature highlights the efficacy of high-frequency stimulation (10 Hz) applied to the hand representation of the primary motor cortex for pain relief (8, 10–18). Focal unilateral stimulation leads to widespread pain relief in these studies (8, 10, 11, 19) thought to result from the non-somatotopic effects of rTMS stimulation (8, 19).

One key consideration in the use of rTMS in SCI is the possibility of interference with ITB pumps used to treat spasticity as a result of electromagnetic interference from the testing equipment. For rTMS, implanted pumps or stimulators within or around the head have been cited as absolute contraindications and below the neck as relative contraindications (20). This is a result of the magnetic field that is produced at the coil during stimulation (21). This magnetic field could potentially interfere with the microprocessor in the intrathecal baclofen pump by causing heating and damage (20). In addition, electromagnetic interference can be caused by other equipment related to data acquisition. However, it is unclear if rTMS does indeed alter the function of an ITB pump placed around the waist (the typical placement of an ITB pump). It is important to consider this question because patients with SCI are frequently administered ITB pumps for spasticity control.

The purpose of this case study was to obtain pain relief by implementing an rTMS treatment protocol in an individual with SCI who is suffering from NP despite the presence of an ITB pump to control spasticity. Our objectives were to: (1) assess pain reduction, both temporary and prolonged, during and following a 6-week intervention, (2) assess the utility of top up sessions following initial treatment to aid in pain relief by rTMS and (3) assess the feasibility of safely conducting rTMS in a patient with an ITB pump.

## Methods

### Experimental protocol

**Figure 1:**
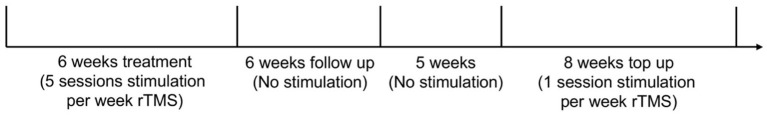
Experimental timeline. The participant took part in 37 total days of stimulation with 44 total administrations of rTMS.

The patient maintained his current medications throughout the duration of the experiment, which included gabapentin, cymbalta, zopiclone, and oxybutynin. The protocol consisted of a treatment period that lasted for 6 weeks with five sessions per week (29 sessions total as the first session began on a Tuesday), followed by a 6-week follow up period. Pain was assessed weekly during the treatment and follow up periods. There was a 5-week period where the participant did not receive treatment and was not tracked for pain. During this time the patient had indicated that there was a relapse in some of the pain that experienced change during the treatment. Top up sessions were implemented as one session per week comprised of two rTMS administrations per day separated by a 2-h period. This occurred for eight consecutive weeks for a total of 15 sessions (rTMS was only administered once on week 8) (Figure 1).

### Electromyography recording

Surface bipolar EMG electrodes were positioned on the skin overlying the right abductor pollicis brevis (APB) muscle. EMG signals were amplified x1000 and filtered from 20–2500 Hz (Intronix Model 2024F; Intronix Technologies Corporation, Bolton, Canada). EMG data were acquired using a Cambridge Electronic Device (Power 1401; Cambridge Electronic Design, Cambridge, UK) and visualized using Signal software (Cambridge Electronic Design).

### Repetitive transcranial magnetic stimulation

rTMS was performed using a Magstim Rapid 2 stimulator (Magstim, Whitland, UK) with 90 mm outer diameter figure-eight coil. The coil was positioned tangent to the scalp at 45° from midline, and the handle of the coil was pointed 5° backward and laterally. rTMS was applied to the motor hotspot of the left motor cortex representation of the APB muscle (10). This target site was digitally registered using Brainsight Neuronavigation software (Rogue Research, Montreal). This location was checked daily to ensure that rTMS was always delivered to the optimal location that elicited motor evoked potentials (MEPs). The rTMS protocol consisted of 2004 biphasic pulses delivered at 10 Hz (8–10) whereby 167 trains of 1.2 s were delivered with a 3s inter-train interval (10). A stimulation intensity of 80% RMT was used (10). The rTMS protocol required ~11.5 min of stimulation applied to the patient for each session of the treatment period. During the top up sessions, the same rTMS parameters were used however the patient experienced two administrations of rTMS per session separated by a 2-h period. In total the patient experienced ~23 min of stimulation during each top up session.

### Management of intrathecal baclofen pump

To ensure no interference and safe baclofen delivery, we consulted with the Medtronic representatives regarding our options. As the ITB pump was positioned on the right side (opposite of the TMS coil) and also considering the distance, chances for interference were considered minimal. For the first week of sessions, we decided to interrogate the ITB pump both before and after the session, in the presence of Medtronic technicians. The study personnel were also trained to reset the pump should it be turned off by the rTMS. As we observed no changes to pump functioning during the first week, we decided to interrogate only on the first session of each week for subsequent treatment sessions. Pump interrogation was only performed during the treatment period and was not performed during the top up sessions.

### Assessments

The primary outcome was related to pain severity. Pain severity was evaluated using the numeric rating scale (NRS) and neuropathic pain scale (NPS). The NRS was scored from 0 to 10 where 0 represents no pain and 10 represents the worst pain possible (22). An NRS was used to quantify pain for each of the four identified pain phenomena. The NPS consists of 10 questions that quantify the severity of NP. Each question is rated from 0 ‘no pain' to 10 ‘most pain imaginable' (23, 24). The score of each question is totaled and presented as a value out of 100. The NPS also has an 11th question that probes the time quality of the pain. The NRS and NPS were recorded at the beginning of the first session each week during the treatment, as well as weekly during the follow up and top up periods.

The secondary outcome measures were the Hamilton Anxiety Rating Scale (HAM-A) (25) and the Hamilton Rating Scale for Depression (HAM-D) (26). The HAM-A is a 14 item questionnaire, with total ranging from 0 to 56 where a score of 14–17 classified as mild, 18–24 moderate, and ≥ 25 severe. The HAM-D is a 21-item questionnaire with the total ranging from 0 to 53. A score of 8–13 is classified as mild, 14–18, moderate, and >19 severe. The HAM-D and HAM-A were recorded at the beginning of the first session each week during the treatment, as well as weekly during the top up period.

## Results

### Pain severity assessed through numeric rating scale

**Figure 2:**
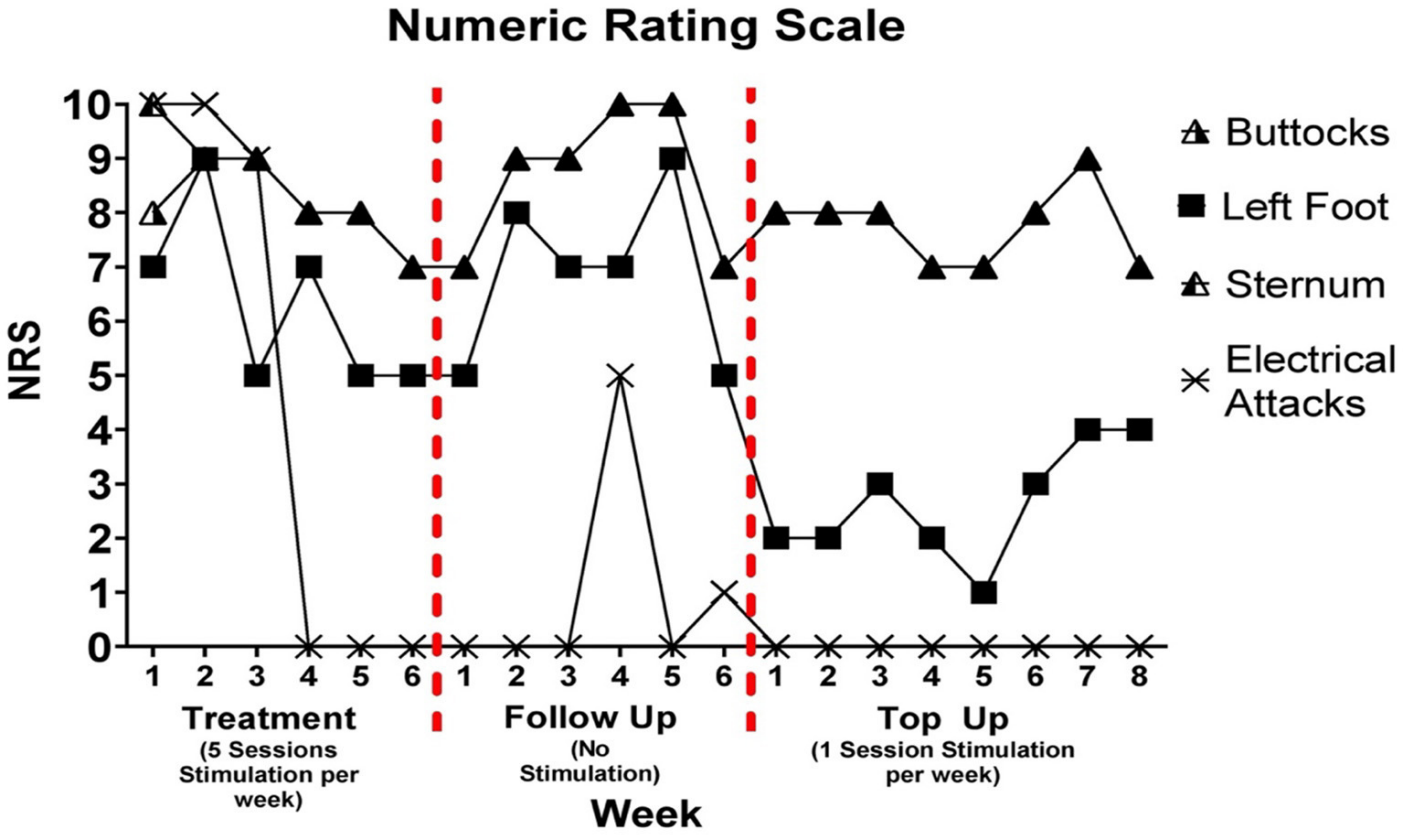
Numeric ratings scale was delivered once per week during treatment, follow up, and top up sessions. There was a 5-week period between follow up week 6 and top up session week 1. It is important to note that pain rating for buttock and sternum differed on week 1 of treatment; however, the rating was the same for both pain locations at each subsequent assessment. A filled in triangle indicates that the pain rating was the same for buttocks and sternum.

Change in pain severity assessed through NRS is presented in Figure 2. During the treatment period, attacks of circulating electrical type pain were abolished following the third week. A 30, 13, and 29% reduction in pain was observed in the sternum, buttocks, and left foot, respectively from week 1 to 6.

During the follow up period, these changes in pain were stable for the first week and then began to increase until they peaked at week 5. A single brief bout of electrical pain with low pain intensity was reported on the 4th and 6th week of the follow up period. A decrease in pain intensity was noted from week 5 to 6 of follow up for left foot, sternum, and buttocks pain types. The patient reported that the frequency of electrical attacks during the 5-week period where tracking did not occur remained at zero.

During the top up sessions, a 13% decrease in pain was observed for sternum and buttocks from week 1 to week 8. There was a 1-point reduction in left foot pain from week 1 to week 5 followed by a three-point increase from week 5 to 8. The frequency of electrical attacks remained at zero during the top up sessions.

### Neuropathic pain scale

**Figure 3:**
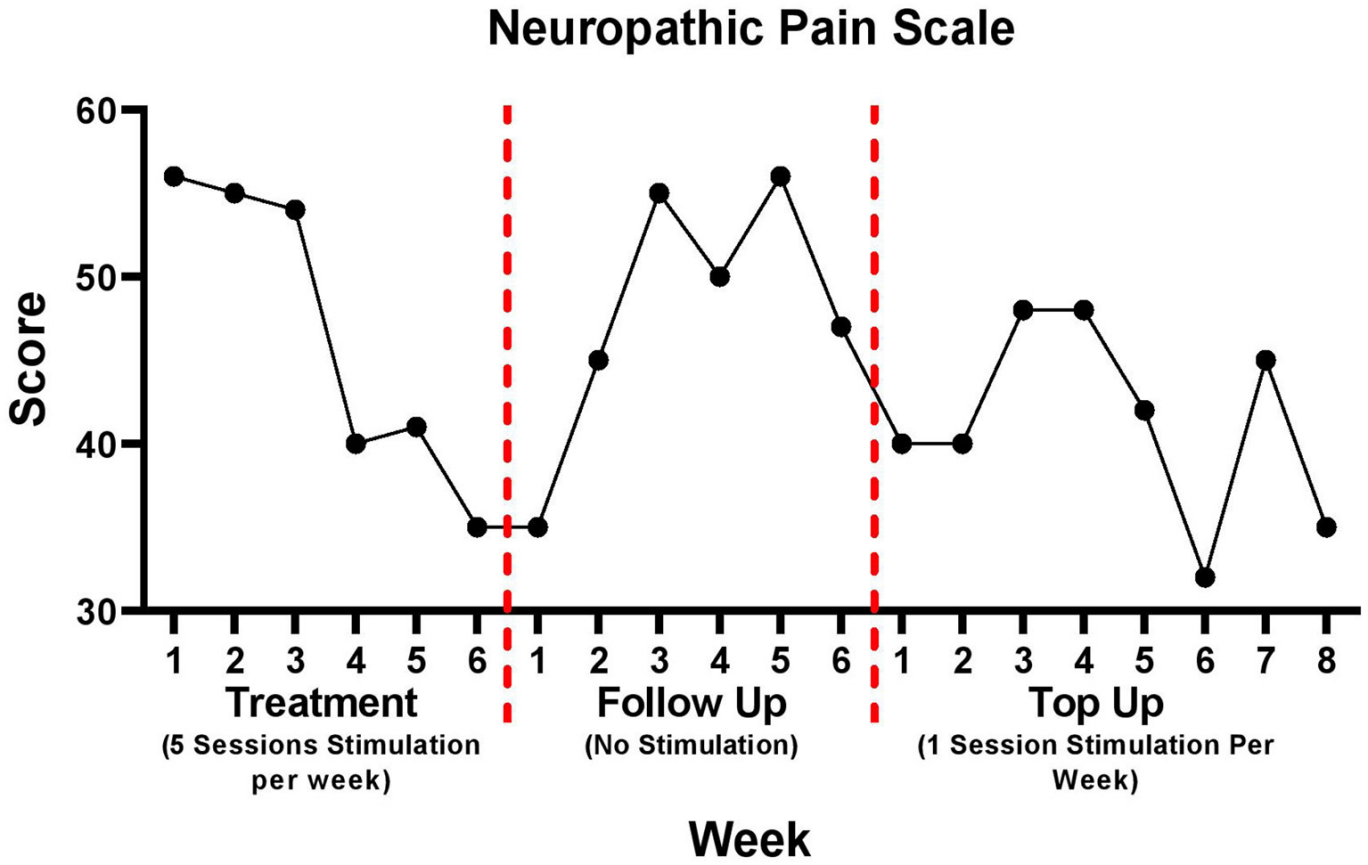
Neuropathic pain scale data for treatment, follow up, and top up sessions. There was a 5-week period between follow up week 6 and top up session week 1.

Changes in NPS are illustrated in Figure 3. Improvements occurred from week 1 of treatment (56/100) to week 6 of treatment (35/100) representing a 38% decrease in NPS scores. During the follow up period, NPS scores increased 34% from week 1 (35/100) to week 6 (47/100). A 13% reduction in pain was observed during the top up period from week 1 (40/100) to week 8 (35/100). The time quality of the pain was recorded as “background pain present all the time, with occasional flare ups”.

###  Depression and anxiety

**Figure 4:**
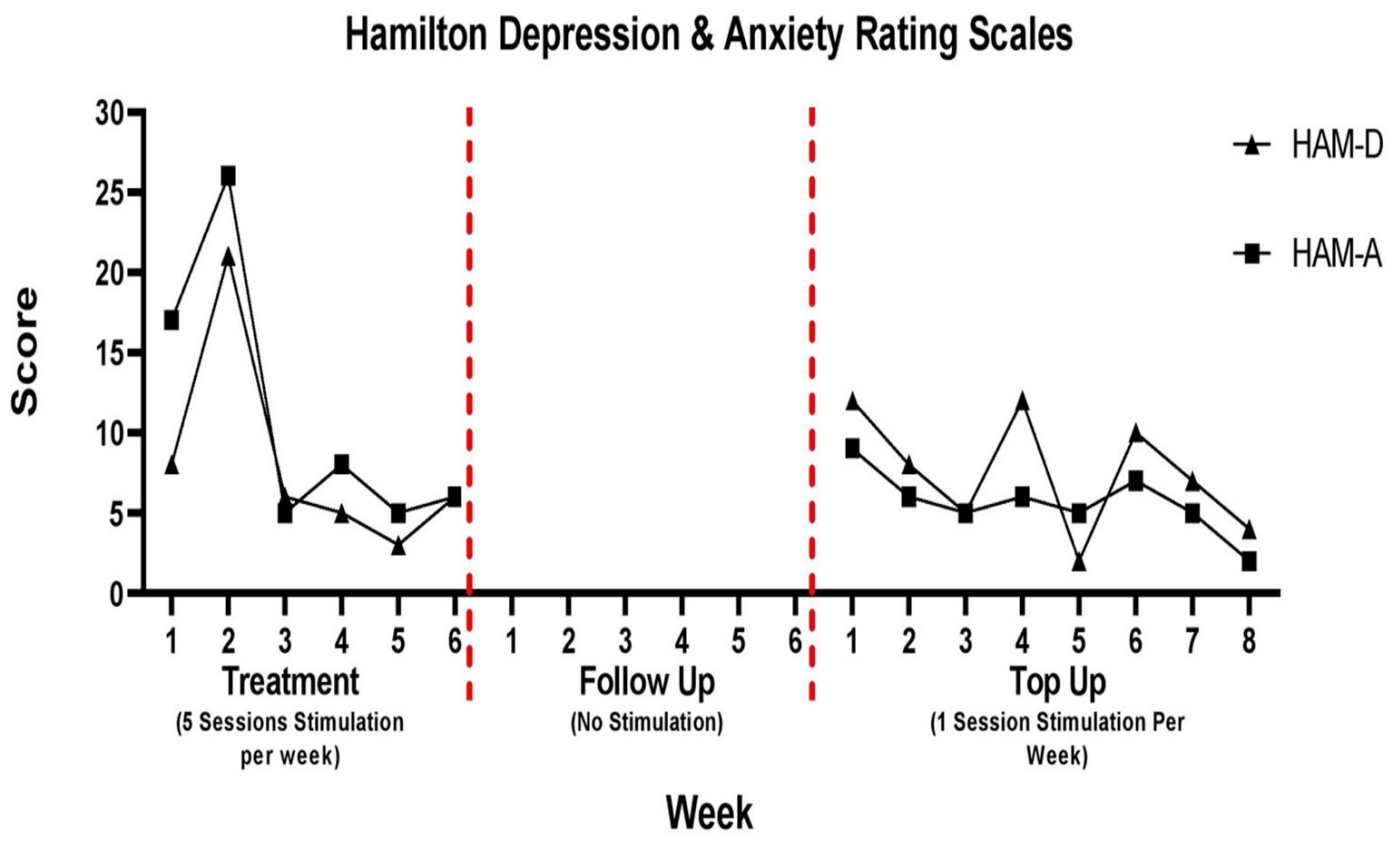
Hamilton Rating Scale for Depression (HAM-D) and Anxiety (HAM-A).

Changes in depression and anxiety scores are presented in Figure 4. During the 6-week follow up period depression and anxiety scores were not tracked. The patient was not coming into the hospital during this time and did not complete the take home questionaries provided to him.

For the HAM-D, there was a spike in depression on week 2 (21/53). There was an overall 25% reduction in depression scores from week 1 (8/53) to week 6 (6/53) of the treatment period. During the top up sessions, the patient depression scores varied week to week, although in some weeks the score was seven, it was always <20. There was a 67% reduction from week 1 (12/53) to week 8 (4/53) of the top up sessions.

For the HAM-A, following the spike on week 2, scores remained stable for the remainder of the treatment period and the patient was classified as having mild anxiety. There was a 65% reduction in anxiety scores from week 1 (17/56) to week 6 (6/56) of the treatment period. This was consistent during the top up period in which the anxiety score remained less than 10 throughout. There was a 78% reduction from week 1 (9/56) to week 8 (2/56) of the top up sessions.

## Discussion

This study describes the use of rTMS as a treatment for unresolved NP following complete SCI in a single patient. The delivery of rTMS did not yield adverse side effects for the participant nor for the function of the ITB pump positioned in the right abdomen. The patient experienced four distinct pain phenomena including pain in the buttocks, sternum, left foot, and electrical attacks. Due to the nature of the different pain phenomena experienced by the patient, the analgesic effects of rTMS were considered separately for each pain type. 10 Hz rTMS applied over the hand representation of the primary motor cortex was effective in reducing the magnitude of pain during the treatment period. The electrical attacks were particularly altered from rTMS.

During the treatment period, there was a 1-point, 3-point, and 2-point reduction in buttocks, sternum, and left foot pain. This decrease began following the third week of stimulation. The magnitude of change observed in the NRS is consistent with previous literature employing rTMS to treat NP. These past studies have reported a 1.5-point decrease in NRS following eight sessions of rTMS (11) and 3-point decrease following 2 weeks of rTMS (10). For a change in pain to be considered ‘clinically important', a reduction in NRS of at least 2 points must occur (27–29). Our findings indicate that not all pain phenomena are equally sensitive to rTMS. This difference may relate to different underlying pathophysiological mechanisms that govern individual pain phenotypes.

The most notable results during the treatment period were the abolishment of electrical attacks that began at approximately week 3 of treatment. Pain attacks, whereby the individual experiences an intense transient bout of pain have been previously cited to occur in individuals with NP (30–32). The characteristics of the pain attacks reported by these studies are consistent with those experienced by the patient in this study, in that the pain is sudden and consists of an electric shock-like feeling. Although pain attacks are a common pain feature of NP, only one study has shown the effects of rTMS on this specific pain type in patients with atypical facial pain, cluster headache, and trigeminal neuropathic pain (33). rTMS delivered 5 days per week for 2 weeks reduced the number of painful attacks per day from 5.6 to 2.3 (33).

Following the treatment period, pain reduction in buttock, sternum, and left foot pain lasted for 1 week. There were also two, short bouts of low intensity electrical attacks. During these attacks, the patient described that the pain began in the left flank but ended before spreading throughout the torso, which was the typical course prior to commencing this study. These findings may have been a result of the complete stop of rTMS sessions, rather than a gradual decrease in frequency over a period of time. Indeed, several studies have highlighted the beneficial effects on the duration of pain relief by gradually decreasing the frequency of rTMS sessions over time following the treatment period (11, 13, 33, 34). These periods are usually referred to as an induction phase, whereby rTMS stimulation is delivered over consecutive days for a set period (treatment), followed by a maintenance period. This ‘weaning' approach may be beneficial for prolonging the analgesic effects of rTMS.

Although a ‘maintenance' period was not used in the current study, it was decided to offer the patient top up sessions as pain relief achieved during the treatment period did not persist for a sustained period of time. The top up sessions performed in this study did not provide clinically important changes for sternum, buttock or left foot type pains. This may have been a result of the length of time between the last session of treatment and the first top up session. Previous work has reported a washout period minimum of 18.3 days after stimulation (34). As a result, it can be inferred that neuroplastic changes that may have occurred from the treatment period were not present during the beginning of the top up sessions. In addition, the quality of evidence of single sessions of rTMS for inducing pain relief is very low (15). This may suggest that the neuroplastic effects of rTMS are cumulative and require consecutive sessions of stimulation to produce meaningful changes. This could explain why pain relief was observed when 5 consecutive sessions were performed each week (treatment) in comparison to one session every week (top up) in this study.

The trends observed in NRS reported pain throughout the study period are comparable to those reported by the NPS. Specifically, as pain decreased during the treatment period as reported through the NRS, so too did the NPS score. Similarly, as pain increased in the follow up period so too did the NPS. These findings are expected as the NPS probes specific characteristics of the overall pain experience. Specifically, the questions of this scale are related to the intensity of pain as well as its characteristics such as how sharp, and unpleasant the pain is. Specific changes noted by the NPS were reduction in skin sensitivity to touch, sharpness of pain, and intensity of surface pain. The NPS can therefore be used to further support the effects achieved through rTMS noted by the NRS (23, 24, 35, 36).

A similar trend in HAM-A and HAM-D scores can be seen during the treatment period, whereby an improvement in anxiety and depressive symptoms occurred after the second week. These findings are consistent with previous literature that have highlighted the beneficial effects of rTMS stimulation for symptoms of NP related anxiety and depression (37). A spike in depression and anxiety scores can be noted on week 2 of the treatment period. After consulting with the patient, this spike seems to be a result of the patient expecting to experience pain relief by that point in the study which was not the case. During the top up period, anxiety and depression scores remained relatively low despite no improvements in pain symptoms. This may indicate that HAM-D and HAM-A measures in this study accurately probed symptoms of anxiety and depression and were not just a reflection of pain improvement. A similar effect was noted by Hodal et al. (37) who found that rTMS had no effect on pain intensity but did lead to improvements in anxiety and depression (37).

The mechanisms that underpin rTMS effects on NP after SCI are not clearly understood. Current evidence suggests the analgesic effects of rTMS may be a result of reinstating intracortical inhibition at the motor cortex as chronic pain is associated with motor cortex disinhibition (38). This may cause descending inhibition from motor cortex to thalamus which projects to the spinal cord (39, 40). Specifically, there is a restoration of GABAergic inhibitory processes (19, 41, 42). High frequency rTMS also causes activation of the endogenous opioid system (40, 43) and activates circuits that project from the motor cortex to structures involved in pain perception such as anterior cingulate and prefrontal cortices (39, 44, 45). This global change induced by rTMS suggests that analgesic effects can be achieved by delivering rTMS to cortical representations of body parts not experiencing pain (8, 46).

rTMS did not cause any interference with the function of the ITB pump for the patient in this study. Although a larger study would need to be conducted to validate this finding, these initial results suggest that rTMS may be safely delivered to patients with ITB pumps. These findings extend upon the work by Klein et al. (20) who suggested rTMS is a relative contraindication for patients with ITB pumps below the neck. Although caution should be taken when administering rTMS to patients with implanted pumps in the abdomen, this should not be considered as an exclusion to participate.

This study did have several limitations that need to be acknowledged. The results of the present study are exploratory and based exclusively on the interpretation of findings from a single patient. In addition, maintenance sessions were not included immediately following the treatment period. The main reason for not including a maintenance period in this study was to better understand the long-term effects of a 6-week treatment period. In addition, the assessments of pain in this study are based on self-reported questionaries which can introduce bias into the data set. Although pain did increase after the first week of follow up, it is important to note that the patient suffered from a urinary tract infection immediately following the treatment period, during the follow up period, which may have inflated the pain scores. The infection was controlled by antibiotics and symptoms subsided following week 5 of the follow up. We cannot rule out however, that the pain relief experienced during the treatment period may have persisted for a longer duration if the urinary tract infection did not occur.

## Conclusion

In conclusion, in this case study we show that rTMS delivered five times per week for 6 weeks to the hand representation of the primary motor cortex was effective in relieving severity of overall NP as reported by the NRS and NPS, during and 1 week following a 6-week treatment period. In addition, this study showed that certain pain phenotypes such as electrical attacks responded more favorably to rTMS stimulation. The duration of pain relief was not long lasting and top up sessions delivered 11 weeks after treatment were ineffective at reducing pain to the level achieved during the treatment period. Maintenance sessions delivered immediately following treatment may aid in prolonging pain relief. Taken together, these findings lend support for the use of rTMS over consecutive sessions to treat patients with refractory NP. Specifically, rTMS may be particularly beneficial for patients who suffer from electrical attacks. Although the analgesic effects achieved during the treatment period are promising, especially those seen for electrical attacks, larger sham-controlled studies are required to elucidate the effect of rTMS on specific NP phenotypes.

## Data availability statement

The raw data supporting the conclusions of this article will be made available by the authors, without undue reservation.

## Ethics statement

The studies involving human participants were reviewed and approved by Hamilton integrated Research Ethics Board (HiREB) affiliated with McMaster University, St. Joseph's Healthcare Hamilton and Hamilton Health Sciences. The patients/participants provided their written informed consent to participate in this study. Written informed consent was obtained from the individual(s) for the publication of any potentially identifiable images or data included in this article.

## Author contributions

SF, HS, and AN contributed to conception and design of the study. SF, RR, and CT performed data collection. SF performed data analysis and wrote the first draft of the manuscript. RR made figures for the manuscript. All authors contributed to the article and approved the submitted version.

## Funding

We thank the Canada Research Chairs program to AN for financial support.

## Conflict of interest

The authors declare that the research was conducted in the absence of any commercial or financial relationships that could be construed as a potential conflict of interest.

## Publisher's note

All claims expressed in this article are solely those of the authors and do not necessarily represent those of their affiliated organizations, or those of the publisher, the editors and the reviewers. Any product that may be evaluated in this article, or claim that may be made by its manufacturer, is not guaranteed or endorsed by the publisher.
